# Structural perspectives on antimicrobial chemokines

**DOI:** 10.3389/fimmu.2012.00384

**Published:** 2012-12-28

**Authors:** Leonard T. Nguyen, Hans J. Vogel

**Affiliations:** Biochemistry Research Group, Department of Biological Sciences, University of CalgaryCalgary, AB, Canada

**Keywords:** chemokine, antimicrobial peptide, chemokine structure, chemokine oligomerization, glycosaminoglycan

## Abstract

Chemokines are best known as signaling proteins in the immune system. Recently however, a large number of human chemokines have been shown to exert direct antimicrobial activity. This moonlighting activity appears to be related to the net high positive charge of these immune signaling proteins. Chemokines can be divided into distinct structural elements and some of these have been studied as isolated peptide fragments that can have their own antimicrobial activity. Such peptides often encompass the α-helical region found at the C-terminal end of the parent chemokines, which, similar to other antimicrobial peptides, adopt a well-defined membrane-bound amphipathic structure. Because of their relatively small size, intact chemokines can be studied effectively by NMR spectroscopy to examine their structures in solution. In addition, NMR relaxation experiments of intact chemokines can provide detailed information about the intrinsic dynamic behavior; such analyses have helped for example to understand the activity of TC-1, an antimicrobial variant of CXCL7/NAP-2. With chemokine dimerization and oligomerization influencing their functional properties, the use of NMR diffusion experiments can provide information about monomer-dimer equilibria in solution. Furthermore, NMR chemical shift perturbation experiments can be used to map out the interface between self-associating subunits. Moreover, the unusual case of XCL1/lymphotactin presents a chemokine that can interconvert between two distinct folds in solution, both of which have been elucidated. Finally, recent advances have allowed for the determination of the structures of chemokines in complex with glycosaminoglycans, a process that could interfere with their antimicrobial activity. Taken together, these studies highlight several different structural facets that contribute to the way in which chemokines exert their direct microbicidal actions.

## Introduction

Chemokines are a superfamily of small globular proteins (8–12 kDa) that play essential roles in both innate and adaptive immunity (Esche et al., 2005; Allen et al., 2007). These ~70 residue proteins are responsible for the trafficking and activation of all leukocytes through interactions with G-protein-coupled chemokine receptors. Additionally, chemokine-mediated cell migration is involved in processes such as tissue development, angiogenesis, cancer progression, and infection (Kiefer and Siekmann, 2011; Lehner et al., 2011; Balkwill, 2012).

**Figure 1:**
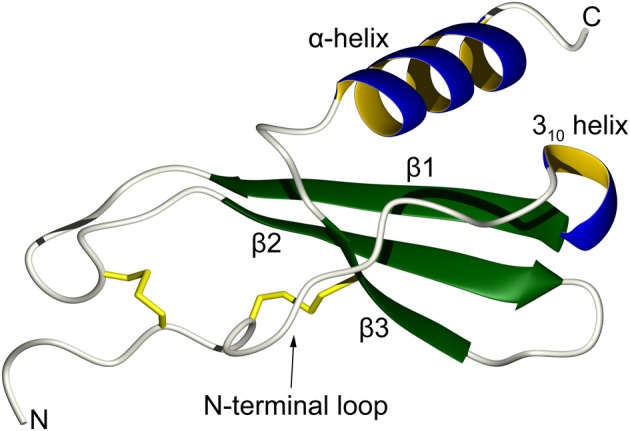
**Topology of a typical chemokine.** The N-terminal loop, which is responsible for receptor recognition, is restrained by two disulfide bonds (in yellow). This is followed by a short turn of a 3_10_ helix that leads to a three-stranded antiparallel β-sheet. The C-terminal α-helix folds over the β-sheet and helps to stabilize the overall tertiary structure.

In order to achieve tight control over the biological activities of the diverse types of leukocytes, there are close to 50 human chemokines that can activate 19 different transmembrane receptors (Allen et al., 2007). Chemokines are usually classified into four categories according to the pattern of several conserved disulfide-bonded cysteine residues near their N-terminal regions: CC, CXC, C, and CX_3_C. Despite large differences in their amino acid sequences, the three-dimensional monomeric structures of chemokine are remarkably similar to each other. They are composed of the following secondary structure elements: an extended N-terminal loop that is important for receptor activation, a central three-stranded antiparallel β-sheet that provides a stable scaffold, and a C-terminal α-helix that stabilizes the overall structure by folding over the small β-sheet (Figure 1) (Fernandez and Lolis, 2002; Allen et al., 2007). Chemokine activity can to some extent be regulated by post-translational proteolytic processing and by chemical modifications that can take place under different physiological and pathological conditions (Wolf et al., 2008; Mortier et al., 2011). Further regulation can occur through modulation of the oligomerization state as chemokines may form homodimers, heterodimers, tetramers, or higher oligomers under various physiological conditions (Weber and Koenen, 2006; Salanga and Handel, 2011). Chemokines also interact with glycosaminoglycans (GAGs), which are themselves a heterogeneous group of polysaccharides that are a part of extracellular proteoglycans, and these interactions further modulate the function, oligomerization state, and localization of chemokines (Proudfoot, 2006).

Following the initial discovery of an antimicrobial chemokine by Dankert and coworkers (Krijgsveld et al., 2000), the majority of human chemokines are now known to play a role as direct broad-range microbicidal agents, an activity that is otherwise primarily associated with antimicrobial peptides (AMPs) (Arias et al., 2012). AMPs are not only another class of bactericidal or bacteriostatic molecules secreted by the innate immune system, but they are also major players in host defense systems (Brown and Hancock, 2006). Thus far, well over a thousand AMPs have been identified (Seshadri Sundararajan et al., 2012). Most AMPs are capable of selectively causing disruptions in the bacterial membrane integrity (Haney et al., 2010), however, the list of possible mechanisms is growing and a peptide may have multiple molecular targets to achieve its antimicrobial activities (Nicolas, 2009; Nguyen et al., 2011a). Although AMPs are often classified in broad structural terms as α-helical, β-sheet or extended, the vast majority display an amphipathic structure in their membrane-bound form that encompasses a hydrophobic and a positively charged surface. Additionally, a few mammalian AMPs have been shown to induce chemotaxis thereby emulating the action of chemokines (Yang et al., 1999; Bowdish et al., 2005; Auvynet et al., 2008).

In a comprehensive study where 30 human chemokines were assayed for bactericidal activity, 18 members were found to have activity against both *Escherichia coli* and *Staphylococcus aureus* (Yang et al., 2003). Most chemokines generally have high isoelectric points around 9.0 or higher, and the high abundance of Arg and Lys residues is thought to be responsible for the antimicrobial activity. Surveying the current literature, only 3 out of 43 chemokines do not have any antimicrobial activity reported, while four others have yet to be assayed (Yung et al., 2011; Arias et al., 2012; Burkhardt et al., 2012). Of the non-antimicrobial chemokines [CC chemokine ligand 3 (CCL3), CCL16, and CCL24], two have particularly high pI's (4.48, 9.86, and 10.76, respectively), therefore overall cationicity cannot be considered as the single reliable predictor of antimicrobial activity. Nevertheless, the high number of antimicrobial chemokines conflicts with some of the negative results reported in the initial study by Yang et al. (2003). This can be reflective of variations in assay media and the different bacterial strains used by various research groups and, occasionally, the variant of the chemokine being studied. In many of the studies mentioned in this article, a chemokine that is considered as non-antimicrobial is only categorized as such in direct testing comparisons to a chemokine that demonstrates high antimicrobial activity, although they may both be active in another assay. Assays are often performed in low salt media and many chemokines can lose their activity at increased salt concentrations (Collin et al., 2008), however, this is not always the case. For example, CCL4 has a low pI of 4.47 and is not antimicrobial in 10 mM NaCl, but it gains growth inhibitory activity against methicillin-sensitive *S. aureus* (MSSA) and methicillin-resistant *S. aureus* (MRSA) at a physiological concentration of 137 mM NaCl (Yung et al., 2011). Additionally, some chemokines have been assayed for antimicrobial activity in a biomatrix that resembles human blood (Yeaman et al., 2007).

Due to their relatively small size, the structures of chemokines can be studied by nuclear magnetic resonance (NMR) spectroscopy in atomic detail, starting with the first chemokine structure reported for CXCL-8/IL-8 (CXC motif chemokine 8/interleukin-8), which was studied as a dimer in solution (Clore et al., 1990). In addition to obtaining detailed three-dimensional structure information, different NMR techniques can be used to study chemokines with regard to their local dynamics, to map out the interfaces between subunits of dimers or higher oligomers, or to study their interactions with GAGs. Although it is outside of the scope of this article, significant advances have also been made recently in the characterization of chemokine-receptor interactions by solution NMR methods (Kofuku et al., 2009; Yoshiura et al., 2010). Here, we will evaluate the various structural properties of chemokines (summarized in Table 1) in an attempt to explain their “moonlighting” function as direct antimicrobial agents.

**Table 1:** **Summary of antimicrobial chemokines discussed in this review**.

**Chemokine**	**Classical nomenclature**	**Structural aspects discussed**	**References**
XCL1	Lymphotactin	Conformational inter-conversion	Tuinstra et al., 2008
CCL5	RANTES	Oligomerization	Wang et al., 2011
CCL13	MCP-4	Antimicrobial fragment (α-helix)	Martinez-Becerra et al., 2007
CCL20	MIP-3α	Antimicrobial fragment (α-helix)	Hasan et al., 2006; Chan et al., 2008
		C-terminal processing	
		Dimerization	
CCL28	CCL28	Disufide reduction	Hieshima et al., 2003; Liu and Wilson, 2010
		Antifungal fragment (α-helix)	
		Mutational studies	
CXCL4	PF-4	Antimicrobial fragment (α-helix)	Tang et al., 2002; Yeaman et al., 2007
		Heterodimerization	
CXCL6	GCP-2	Antimicrobial fragment (β-sheet)	Linge et al., 2008
		Membrane-induced conformational change	
CXCL7	PBP, CTAP-3, NAP-2, TC-1	Antimicrobial fragment (N-term region)	Krijgsveld et al., 2000; Tang et al., 2002; Kwakman et al., 2011; Nguyen et al., 2011b
		C-terminal processing	
		Local dynamics	
		Mutational studies	
		Membrane-induced conformational change	
		Disulfide reduction	
		Dimerization	
		Heterodimerization	
CXCL8	IL-8	Antimicrobial fragment (α-helix)	Bjorstad et al., 2005; Pichert et al., 2012
		GAG interactions	
CXCL9	MIG	Antimicrobial fragment (α-helix)	Egesten et al., 2007, 2009
		Disulfide reduction	
CXCL10	IP-10	C-terminal processing	Hensbergen et al., 2004
CXCL12	SDF-1α	Oligomerization	Murphy et al., 2010; Laguri et al., 2011
		GAG interactions	

## Chemokine-derived antimicrobial fragments

The chemokine CCL20/MIP-3α (macrophage-inflammatory protein-3α) is functionally similar to the β-defensin AMPs as they can both activate the same chemokine receptor, CCR6 (Yang et al., 1999), and they are both highly antimicrobial. Structurally, these proteins have an antiparallel three-stranded β-sheet in common. It has been suggested that the host defense peptide groups of chemokines and cysteine-containing AMPs such as the defensins may share a unifying structural signature termed the γ-core motif (Yount et al., 2007). CCL20 can be hydrolyzed at positions 52 or 55 by cathepsin D, a protease involved in tumor progression (Hasan et al., 2006). The C-terminal fragment, comprising the α-helix of the intact protein, retains antimicrobial activity while the N-terminal portion (residues 1–52) that includes the entire β-sheet is inactive. The C-terminal peptide of CCL20 is very cationic and has similar properties compared to canonical α-helical AMPs such as magainin (Haney et al., 2009). It is unstructured in aqueous solution and requires a membranous environment to fold into a well-defined amphipathic helix that resembles its conformation within the parent chemokine. This potent chemokine-derived AMP preferentially disrupts anionic model membranes over zwitterionic ones (Nguyen et al., 2010a). These two model membranes are frequently used to represent bacterial and eukaryotic membranes, respectively (Lohner, 2001). Bacterial membranes are rich in phospholipids with phosphatidylglycerol headgroups, which are negatively charged, while the outer leaflet of eukaryotic cells is usually made up of phosphatidylcholine headgroups, which have no net charge.

Peptides covering the entire amino acid sequences of CCL13 and mammalian CXCL4/PF-4 (platelet factor 4) were assayed for antimicrobial activity (Martinez-Becerra et al., 2007; Yeaman et al., 2007). In both cases, the most active fragments covered the α-helices of the chemokines and these had comparable potency to the native proteins. Similar to the CCL20-derived peptide, these peptides include many cationic residues and they seem to permeabilize bacterial membranes to cause notable morphological changes. Interestingly, in the case of CXCL8, the C-terminal α-helix is similar in sequence to a bactericidal peptide from *Helicobacter pylori* (Bjorstad et al., 2005). The peptide corresponding to this region can be released via acid hydrolysis and possesses antibacterial activity that is absent in full-length IL-8. This activity increases at lower pH levels and is attenuated by high salt concentrations. The structure of the peptide bound to SDS micelles was solved using NMR spectroscopy, showing an amphipathic helix where the cationic face, composed of five Lys or Arg side chains, is partially disrupted by the presence of three negatively charged Glu side chains (Bourbigot et al., 2009). Solid-state NMR experiments and functional leakage assays indicate that this peptide may not be able to cause significant membrane disruption (Bourbigot et al., 2009; Nguyen et al., 2010a).

However, the antimicrobial activity of chemokines is not always concentrated in the C-terminal helical region. When separated, the N-terminal 50 residues of CXCL6/GCP-2 (granulocyte chemotactic protein 2) is ten times more bactericidal than its 19 C-terminal residues despite the latter having a higher pI (Linge et al., 2008). However, neither peptide is as active as the parent chemokine. The β-sheet containing N-terminal fragment disturbs anionic membranes to cause liposome leakage levels similar to intact CXCL6 while the C-terminal peptide is not membrane disruptive (Linge et al., 2008).

**Figure 2:**
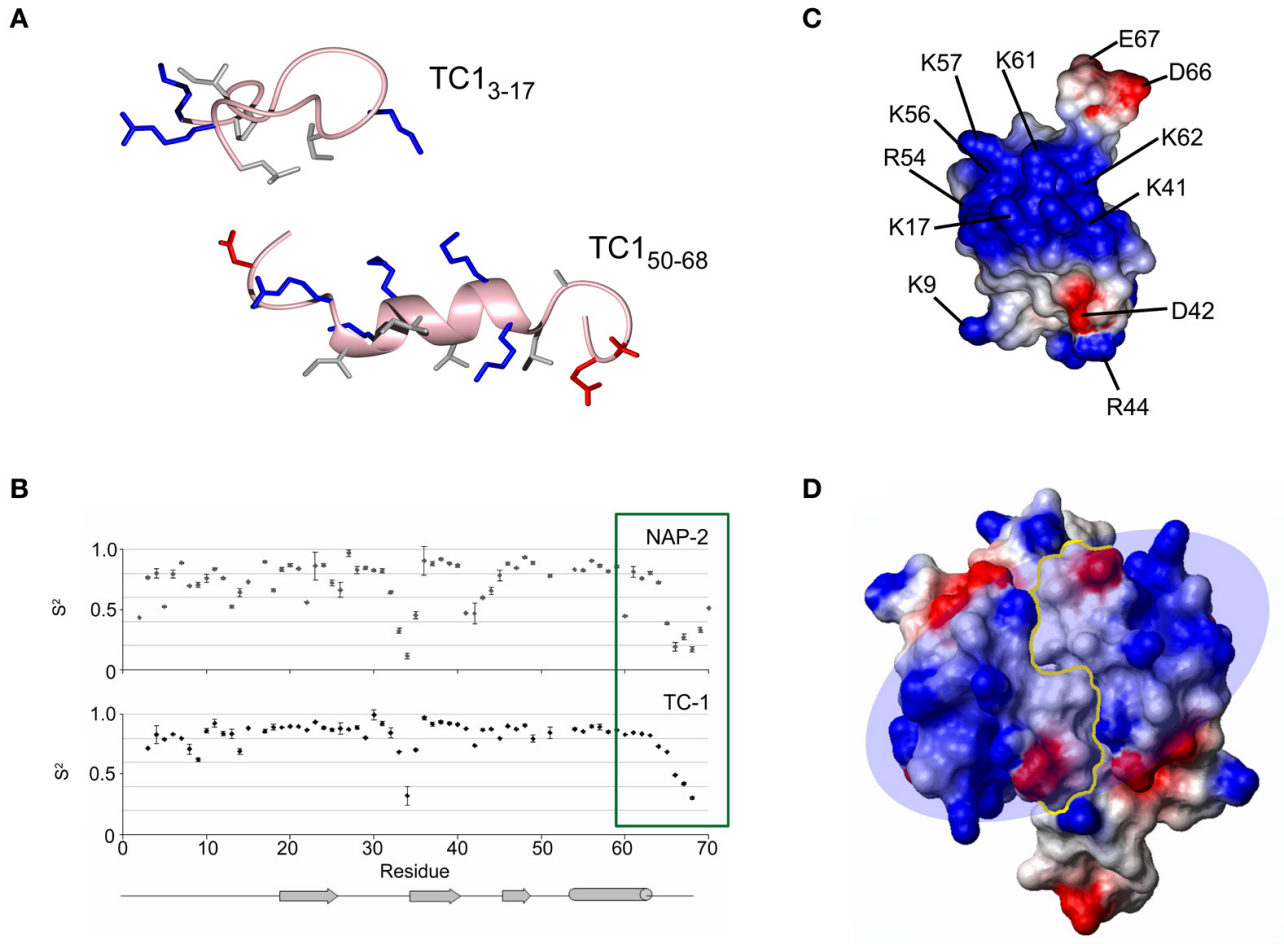
**Different ways in which the structure of the antimicrobial chemokine thrombocidin-1 (CXCL7/TC-1) has been studied by NMR spectroscopy. (A)** Membrane-bound conformations of two peptides from different regions of TC-1 showing some antimicrobial activity. **(B)** Model-fitted order parameters from NMR relaxation experiments comparing the local dynamics of TC-1 and NAP-2. The C-terminal tail of NAP-2 becomes less flexible in its final residues, in contrast to TC-1. **(C)** Electrostatic surface diagram showing the large “positive patch” of TC-1. **(D)** Electrostatic surface diagram showing the extension of the positive patch created through TC-1 dimerization. Panels **(B)** and **(D)** reprinted with permission from Nguyen et al. (2011b).

A set of overlapping 15-mer peptides covering the amino acid sequence of TC-1 (CXCL7/thrombocidin-1), a bactericidal chemokine released from thrombin-activated blood platelet granules, have been assayed for antimicrobial activity (Kwakman et al., 2011). While the peptide corresponding to the C-terminal α-helix displays mild activity, the most potent peptide corresponded to residues 3–17 of TC-1, which are situated in the extended N-terminal fragment and the 3_10_ helical turn preceding the β-sheet. This was unexpected, given that this peptide is not as cationic nor is it more hydrophobic than the 15-residue sequence encompassing the C-terminal α-helix. Interestingly, the peptide has a distinct activity profile compared to TC-1: while they are both equally potent against *Bacillus subtilis*, the peptide is more antifungal against *Cryptococcus neoformans*, and intact TC-1 is more active against *E. coli* and *S. aureus*. This suggests that the peptide and intact TC-1 may employ different mechanism(s) of antibacterial action. NMR spectroscopy experiments show that, in a membranous environment, the N-terminal peptide folds into an extended amphipathic conformation and does not resemble the α-helix that is normally associated with many AMPs of this size (Figure 2A). In the native TC-1 chemokine, the region represented by the peptide has two disulfide bonds that restrain it to the rest of the protein.

## Structural determinants for antimicrobial activity

TC-1 is found in platelet α-granules and it is an antimicrobial derivative of NAP-2 (CXCL7/neutrophil-activating peptide 2) that has been truncated by two C-terminal amino acids, Ala69, and Asp70 (Krijgsveld et al., 2000). NAP-2 itself is the mature product of several N-terminal truncations from PBP (CXCL7/platelet basic protein) (Brandt et al., 2000). Peptides encompassing the α-helical regions of TC-1 and NAP-2 are inactive as antimicrobials, however, their structures solved in a membrane-mimetic environment may help to explain the difference in the activity of these chemokines (Nguyen et al., 2010a). NAP-2 contains four negative charges in its last five residues, and this tail folds back onto the α-helix via electrostatic interactions. This would effectively mask some of NAP-2's cationic surface and would reduce its affinity for negatively charged bacterial membrane surfaces. The last four residues of NAP-2 were not included in the X-ray crystal structure due to poorly defined electron density in this region (Malkowski et al., 1995). NMR relaxation experiments reveal that, although the last five residues are overall quite flexible compared to the rest of NAP-2, the final two residues become more motionally restricted than the residues immediately preceding them (Figure 2B) (Nguyen et al., 2011b). By comparison, the TC-1 backbone continually increases in flexibility toward the end of its sequence and the C-terminus was not seen to fold back over the α-helix in the corresponding peptide. Two additional natural isoforms featuring further C-terminal truncations, removing the last four and seven residues of NAP-2, respectively, have been identified previously (Ehlert et al., 1998). These variants are more potent as neutrophil activators and the removal of more negative charges would be expected to further improve the antimicrobial activity of TC-1, although this has not yet been tested experimentally. The functional activation of NAP-2 in platelets represents a unique case because no other chemokine post-translational processing events have been found to increase the antimicrobial activity (Wolf et al., 2008). In other instances where proteolytic cleavage occurs at the C-terminus, positively charged residues are lost from chemokines that are already microbicidal (Davis et al., 2005; Mortier et al., 2008; Denis et al., 2012). For example, CXCL10/IP-10 (interferon γ-induced protein-10) retains its activities against *E. coli* and *Listeria monocytogeneses* in spite of the loss of two positively charged residues, in a reaction that is catalyzed by the proprotein convertase furin (Hensbergen et al., 2004).

It is possible that the longer C-terminus of NAP-2 can also make electrostatic contacts with other positively charged residues beyond the α-helix. Upon examination of the charge distribution of the TC-1 surface, there is a “positive patch” on the protein surface that becomes evident (Figure 2C). This area features five Lys/Arg side chains from the α-helix and also includes Lys17 in the turn between the N-terminal segment and the first β-strand and three residues (Lys41, Arg44, and Lys45) in the turn between the second and third β-strands (Kwakman et al., 2011). Lys-substituted mutants to augment the positive patch have shown that the inhibitory activity of TC-1 against *B. subtilis, S. aureus*, and *E. coli* can be improved upon. However, these substitutions either had little effect on or abolished the anti-fungal activity.

The positive patch of TC-1 may undergo some rearrangement given that, upon binding to negatively charged membranes, the structure of TC-1 takes on a higher α-helical content as measured by circular dichroism (CD) spectroscopy (Kwakman et al., 2011). The killing kinetics of TC-1 show that it acts within 10 min (Krijgsveld et al., 2000), which is fast enough to suggest that it may perturb the bacterial membrane. However, TC-1 is incapable of dissipating the membrane potential of a *Lactococcus* species (Krijgsveld et al., 2000). CD spectra of intact CXCL6 show that this chemokine also becomes more α-helical upon binding to a membrane surface and, unlike TC-1, CXCL6 is membrane disruptive (Linge et al., 2008). The determination of a high resolution structure of a membrane-bound chemokine by NMR spectroscopy would therefore be of great help in understanding their antimicrobial behavior.

The disulfide reduced form of TC-1 is almost equally effective as an antimicrobial as native TC-1 and it adopts a similar membrane-induced structure despite the loss of secondary and tertiary structure in aqueous solution (Kwakman et al., 2011). Although disulfide bonds are usually essential for retaining chemokine structure in aqueous solution and for maintaining their cell signaling functions (Rajagopalan and Rajarathnam, 2006), linearized analogs of chemokines such as CXCL9/MIG (monokine-induced by γ-interferon) and CCL28 also retain antimicrobial activity (Egesten et al., 2009; Liu and Wilson, 2010). Disulfide bonds are not required for the activity of several AMPs either (Ramamoorthy et al., 2006; Dawson and Liu, 2010); in fact, the human β-defensins-1 and -3 (HBD1 and HBD3) become more active upon linearization (Wu et al., 2003; Schroeder et al., 2011). However, *in vivo*, linear AMPs can be easily degraded in human serum and folded chemokines would be more stable under these conditions (Nguyen et al., 2010b).

CCL28 is a broad-spectrum antimicrobial chemokine that is expressed in mucosal tissues and it can be recovered in high amounts from saliva and milk (Hieshima et al., 2003). The three dimensional structure of the 108-residue long CCL28 has yet to be determined, however, the solution structure of human CCL27, a related 88-residue long chemokine, can provide some information (Jansma et al., 2010). CCL27 has the typical 3-stranded β-sheet followed by an α-helix that runs from Pro59 to Arg70, after which the remaining 18 residues are disordered. The C-terminal region of CCL28 is an important determinant for its antimicrobial activity. The corresponding disordered portion beyond the α-helix in CCL28 would encompass 26 residues, and this region has high sequence similarity to histatin-5 (His-5). His-5 is an antifungal His-rich peptide produced in saliva (Tsai and Bobek, 1998). In aqueous solution, His-5 is unstructured and in the presence of a membranous environment, an α-helical structure is induced (Raj et al., 1998). However, it should be noted that His-5 seems to act intracellularly following membrane translocation (Jang et al., 2010). A CCL28-derived peptide composed of the His-5-like sequence loses activity against five different bacterial strains compared to the parent CCL28 chemokine and, like His-5, it is mostly an antifungal agent (Hieshima et al., 2003). His-5 is internalized into *C. albicans* cells following translocation. However, its killing mechanism as well as the role of its ability to bind divalent cations such as Zn^2+^ and Cu^2+^ remain unclear (Gusman et al., 2001; Jang et al., 2010). Whether the His-rich tail of CCL28 can similarly coordinate metal ions remains to be determined.

Truncation mutants of murine CCL28 (mCCL28) indicate that, although optimal activity is retained only in the full chemokine, it can tolerate the loss of 18 residues in its C-terminus before it loses significant bactericidal activity (Liu and Wilson, 2010). These authors also identified a sequence at positions 85–89 (RKDRK) that is essential for the antimicrobial activity. However, in amino acid sequence alignments, this particular motif is noticeably absent in mice and many other mammals. The corresponding motif in CCL28 from chimpanzees, monkeys and humans (KRNSN) loses one positive charge, yet the latter homolog is more antimicrobial than mCCL28 (Hieshima et al., 2003). The C-terminal half of mCCL28, comprised of the putative α-helix and the long disordered tail, when assayed by itself is not as microbicidal as full-length mCCL28 (Liu and Wilson, 2010). Because mCCL27 is a non-antimicrobial chemokine that has high sequence homology to mCCL28, a construct was made combining the N-terminal half of mCCL27 and the C-terminal half of mCCL28, and this chimeric protein was able to recover the full activities of mCCL28. The C-terminal region of mCCL28 was also combined with the N-terminal half of mCCL5, an unrelated chemokine with low antimicrobial activity, and this led to an interesting activity profile. Compared to mCCL28, the chimera had diminished activity against Gram positive *S. aureus*, but it had retained full activity against Gram negative *P. aeruginosa* (Liu and Wilson, 2010). Therefore, the two halves of mCCL28 contribute in different proportions to its activity against different species.

## The role of dimerization and oligomerization

3D structures of chemokines are often solved by X-ray crystallography or by NMR spectroscopy as dimers or tetramers (Figure 2). This may be due to the high protein concentrations required for such studies. The oligomerization dissociation constants of chemokines are usually in the micromolar range which would therefore lead to expectations that they are monomeric in serum where their concentrations are typically in the nanomolar range (Fernandez and Lolis, 2002). However, the self-association equilibria can be shifted toward the dimerization state by changes in pH, salt concentration and the presence of anions such as phosphate, sulfate and by the presence of GAGs (Mayo and Chen, 1989; Veldkamp et al., 2005). Pulsed field gradient NMR experiments measuring molecular diffusion rates can give indications as to the oligomerization state of a chemokine under different conditions. In CXCL12/SDF-1α (stromal cell-derived factor-1α) and CCL20, the dimerization is influenced by the protonation state of specific histidine residues, His28 in the first β-strand of CXCL12 and His40 in the second β-strand of CCL20. At pH values below the pKa of these residues, the charged protonated His residues repel each other and this in turn breaks the intermolecular contacts and the monomeric state is favored (Veldkamp et al., 2005; Chan et al., 2008). In contrast, interactions with highly negatively charged GAGs usually promote dimerization and may help in establishing chemokine gradients toward sites of infection (Lortat-Jacob, 2009). Receptor activation can be demonstrated for most monomeric chemokines *in vitro*, however, oligomeric forms are also functionally important (Salanga and Handel, 2011). For example, ^15^N-^1^H heteronuclear nuclear Overhauser effect (NOE) and chemical shift perturbation mapping from NMR experiments show that the monomeric form of CXCL12 makes specific contacts with the receptor CXCR4 that are lost after it dimerizes (Drury et al., 2011). However, both forms interact with CXCR4 and cause distinct biological events in colorectal carcinoma cells. The receptor is a constitutive homodimer *in vivo*, therefore the state of its single- or double-ligand occupancy can lead to the activation of different intracellular signaling cascades.

The oligomerization of AMPs is thought to markedly influence their activity, especially concerning membrane disruption (Mihajlovic and Lazaridis, 2010). Disulfide-linked dimeric analogues of α-helical peptides such as magainin usually have improved activity (Tencza et al., 1999; Dempsey et al., 2003), while β-sheet peptides such as the defensin human neutrophil protein-1 (HNP-1) and protegrin-1 have to dimerize to form membrane pores, the latter assembling into a β-barrel-like pore (Mani et al., 2006; Zhang et al., 2010). Furthermore, the higher tendency of HBD-3 to dimerize compared to HBD-1 and HBD-2 is thought to contribute to its higher overall activity and to its salt-resistant antimicrobial properties (Schibli et al., 2002).

It therefore seems likely that the oligomerization of chemokines would also enhance their antimicrobial potential. The dimerization of chemokines may extend their cationic surface across a larger continuous area to facilitate interactions with the negatively charged bacterial surfaces. Apart from the unmasking of the positive patch from the shorter tail of TC-1 as discussed above, this platelet chemokine also has a higher tendency to dimerize compared to NAP-2 and this could further contribute to the differences in their activities (Figure 2D) (Nguyen et al., 2011b). Amongst the closely related monocyte chemoattractant proteins (CCL2/MCP-1, CCL8/MCP-2, and CCL7/MCP-3), CCL7 has the lowest propensity to form dimers and, despite having the highest pI among them, it also has the weakest antimicrobial activity (Kim et al., 1996). However, the tendency to dimerize is not always a requirement for microbicidal chemokines. For example, CCL1/I-309 and CCL15 are known to be monomeric at concentrations up to 2 mM yet they do display some antimicrobial activity (Sticht et al., 1999; Keizer et al., 2000).

**Figure 3:**
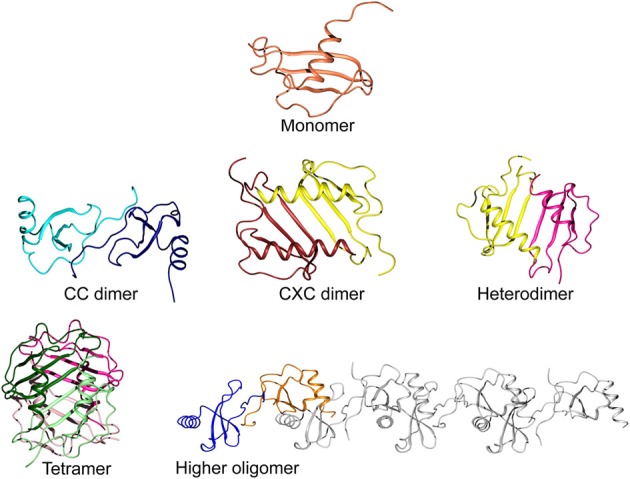
**Levels of oligomerization in chemokines.** A typical chemokine monomeric unit is shown at the top (CCL20; PDB ID 2JYO). The CC-type dimer is formed from contacts between the N-terminal fragments of each subunit to create an elongated shape (CCL2 homodimer; PDB ID 1DOM). The CXC-type dimer is created by the continuation of the β-sheets via their first strands and a small reorientation of the α-helices, running anti-parallel to each other (CXCL8; PDB ID 1IL8). Heterodimerization is also possible, modeled here from two subunits of CXCL8 and PF-4 (PDB ID 1IL8 and 1RHP, respectively). The common tetramer organization of chemokines is shown for CXCL4, colored to highlight their configuration as a dimer of dimers (PDB ID 1RHP). A less common form of higher oligomerization is represented by CCL5 in a linear polymeric chain of repeating dimer units (PDB ID 2L9H).

Most CC chemokines self-associate through the formation of a small antiparallel β-sheet between the N-terminal residues of both subunits, resulting in an elongated structure (Figure 3). Generally, CXC chemokine dimers are more compact with the main contacts formed between the first β-strands of each subunit and a small reorientation of the α-helices (Chan et al., 2008; Gandhi and Mancera, 2011). Given these two main patterns of quaternary structure, a few chemokine pairs have a preference for heterodimerization over homodimerization, which can add another layer to the regulation of their biological functions (Nesmelova et al., 2005; Weber and Koenen, 2006). Such interactions may also promote their antimicrobial activities, and the synergistic bactericidal effect of the platelet chemokines CXCL4 and CTAP-3 (CXCL7/connective tissue-activating peptide 3) against *E. coli* are likely influenced by their heterodimerization (Tang et al., 2002; Nesmelova et al., 2008).

**Figure 4:**
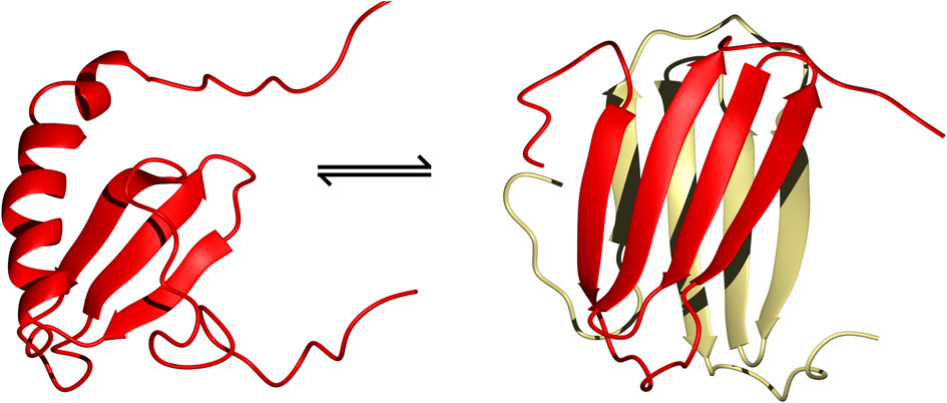
**The two structural folds adopted by XCL1 that co-exist in equilibrium with each other under physiological conditions (Tuinstra et al., 2008).** On the left is the typical chemokine fold of lymphotactin and on the right is the alternate all β-sheet conformation which readily dimerizes to form a head-to-tail β-sheet sandwich (PDB ID 1J9O and 2JP1, respectively).

XCL1/Lymphotactin is a rather unique case where the chemokine structure rapidly interconverts between two unrelated conformations *in vivo* (Figure 4) (Tuinstra et al., 2008). These states can be stabilized by carefully controlling the temperature and salt concentration for structure determination by NMR spectroscopy. In the low temperature high salt condition, the typical chemokine fold of a three-stranded β-sheet followed by an α-helix dominates as a monomer. This form is functional for receptor binding, but does not interact with GAGs. In the high temperature low salt condition, XCL1 rearranges itself into a novel protein fold of a four-stranded antiparallel β-sheet sandwiched with another subunit as a head-to-tail dimer. Using isotope-filtered NOESY NMR experiments on a mixture of labeled and unlabeled XCL1, intermolecular contacts could be observed to identify the dimer interface. This alternate conformation does not interact with the XCR1 receptor. Instead, it clusters the acidic residues of the monomer subunits to one end of the dimer interface, leaving a large surface of positively charges at the opposite end that can interact with GAGs or bacterial membranes (Tuinstra et al., 2008).

Higher order homo-oligomers of chemokines often take the shape of a globular complex. For example, the tetramers of NAP-2 and CXCL10 have been described as dimers of β-sheet dimers (Malkowski et al., 1995; Swaminathan et al., 2003). For CCL5, however, a different form of oligomerization has been observed through the integrated use of various structural techniques (Wang et al., 2011). The relative orientations of the monomer subunits were determined by measuring NMR residual dipolar couplings, while the interfacial contacts were identified by NMR cross-saturation and mass spectrometry footprinting experiments. Also, small angle X-ray scattering results were used to restrict the overall shape of the oligomer. The final model of the aggregate shows repeating units of CCL5 dimers assembling into a linear polymeric chain that may be able to accommodate simultaneous interactions with the receptor as well as with a long GAG molecule. X-ray crystallography data show that similar linear structures can be formed by CCL3/MIP-1α, CCL4/MIP-1β, and CXCL12 (Murphy et al., 2010; Ren et al., 2010). While CCL3 and CCL4 have little to no antimicrobial activity due to their low net charge at neutral pH, the polymerization of the antimicrobial CXCL12 and CCL5 chemokines may make them less susceptible to secreted bacterial proteases.

## Interactions with glycosaminoglycans and other modulatory molecules

Glycosaminoglycans are heterogeneous unbranched polysaccharides with high charge densities, usually coming from sulfate or carboxylate groups that are present in the extracellular matrix as proteoglycan attachments or as freely circulating molecules. GAG binding to chemokines typically promotes their oligomerization and their retention on cell surfaces, which can create high local chemokine concentrations at sites of infection to exert their antimicrobial effects (Proudfoot, 2006). On the other hand, such interactions should diminish the activity of microbicidal chemokines given that the negatively-charged GAGs and bacterial membrane surfaces would compete for the same cationic surfaces on the proteins. In fact, secreted proteinases from the pathogens *P. aeruginosa, E. faecalis*, and *S. pyogenes* can digest proteoglycans, thereby releasing dermatan sulfate which can bind to and inactivate the bactericidal activity of HNP-1 (Schmidtchen et al., 2001). Similar interactions are also possible with HBD-2, where the type of GAG involved in the interaction can promote the presence of either the monomeric or the dimeric form of the peptide (Seo et al., 2010).

A common sequence recognition motif for GAGs such as heparan sulfate or heparin is BB*X*B, where B is a basic amino acid (Hileman et al., 1998). Interacting residues have been identified via mutational studies, and the location of this motif varies between different chemokines. Binding sites appear at the turn before the first β-strand in CXC12; the turn between the second and third strands in CCL7, CCL3, CCL4, and CCL5; and the α-helix in CXCL8 (Salanga and Handel, 2011). The heparin binding interface for CXCL11/I-TAC (interferon-inducible T-cell α chemoattractant) also involves a cluster of positively charged residues at the C-terminal helix (^57^KSKQAR^62^), however, Lys17 is also an important residue (Severin et al., 2010). These residues are not directly involved in receptor binding as a mutant substituting the cationic residues to alanine is still capable of receptor activation and inducing cell migration *in vitro*. However, the inability to cause these events *in vivo* demonstrates that GAG binding is necessary for its physiological function. Affinities for the GAG interaction can vary from K_D_'s in the nanomolar range for the tight binding of CXCL11 to the mid-micromolar range for many other chemokines (Severin et al., 2010; Laguri et al., 2011).

**Figure 5:**
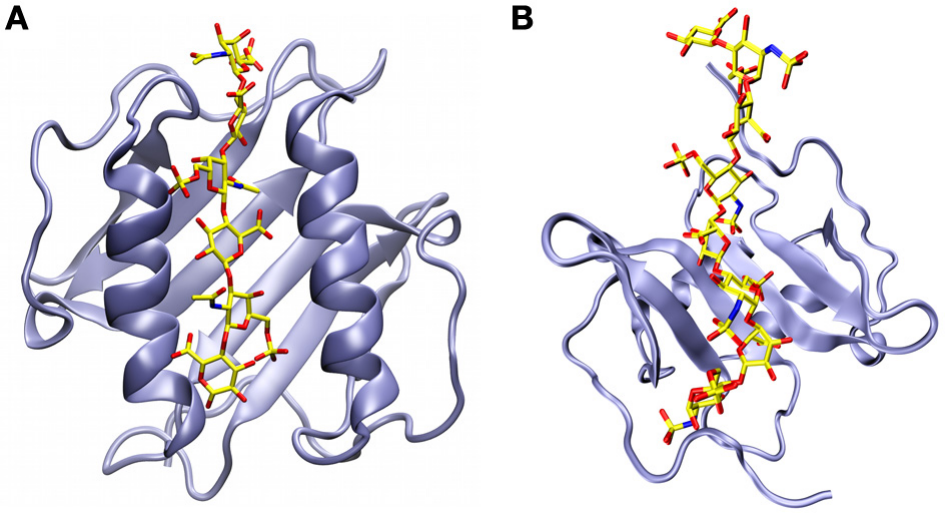
**Structural models of chemokine dimers bound to glycosaminoglycans. (A)** A chondroitin-6-sulfate hexasaccharide sandwiched between the anti-parallel α-helices of the CXCL8 dimer (Pichert et al., 2012). **(B)** A heparan sulfate octasaccharide analogue docked onto the β-sheet of the CXCL12 dimer on the face of the protein that is opposite to where the α-helices are located (Laguri et al., 2011).

GAG-bound chemokine complexes are difficult for high resolution structural studies due to the conformational plasticity of the GAGs and the aggregative tendencies of these mixtures, however, there have been some recent successes. In NMR experiments, the backbone amide peaks of CXCL8 could be followed when hexamers of four different types of GAGs were titrated into separate samples (Pichert et al., 2012). No major chemical shift perturbations were seen in titrations with hyaluronan, which has one carboxylate group in every repeating disaccharide unit, indicating that no major structural changes take place in CXCL8. Chemical shift perturbations were seen, however, in titrations with dermatan sulfate, chondroitin-4-sulfate and chondroitin-6-sulfate. The areas affected involved residues 15–45, covering the end of the N-terminal fragment and the first two β-strands, and most significantly residues 57–77 in the α-helix. In addition to causing large shifts for the basic residues in the α-helix, the movement of the Glu75 amide peak reflects an electrostatic repulsion between this residue and the sulfate groups. Molecular docking of a chondroitin-6-sulfate hexasaccharide onto the CXCL8 dimer shows that the GAG molecule can become sandwiched and act as a bridge for the α-helices of the two subunits that run antiparallel to each other (Figure 5A).

Using a ^13^C-labeled heparan sulfate analog, Laguri and coworkers were also able to obtain structural information for both partners in the complex of CXCL12 bound to a GAG (Laguri et al., 2011). NMR chemical shift perturbations confirmed that Arg12 and Lys24 are the basic residues most involved in the interaction. This and other shift perturbation information was fed along with NMR saturation transfer difference results into Haddock, a data-driven molecular docking program (Dominguez et al., 2003). The resulting energy minimized structures show quite a different picture from the CXCL8 complex, with the octameric GAG now running across the β-sheet of the CXCL12 dimer on the opposite face of the α-helices (Figure 5B) (Laguri et al., 2011).

In addition to GAG-releasing proteinases, some bacteria can secrete proteolytic enzymes that directly modulate chemokine activities. Enzymes secreted by different bacterial sources can cause different changes in a chemokine's range of activity. For example, the streptococcal cysteine proteinase SpeB is a virulence factor released by *S. pyogenes* that degrades several AMPs and several chemokines including CXCL10, CCL5, CCL20, and XCL1 (Egesten et al., 2009). However, it has no activity toward CXCL8 and CCL5. The 103 a.a. CXCL9 is partially digested by SpeB and SufA, a proteinase released by the opportunistic pathogen *Finegoldia magna*. CXCL9 is attacked at both ends, losing a portion of the extended N-terminal loop and residues past the α-helix at the C-terminus. The SpeB-digested CXCL9 fragment loses its chemotactic activity, but it retains activity against *S. pyogenes*. The SufA-digested CXCL9 fragment loses activity against *F. magna*, however, remains antimicrobial towards *S. pyogene*. SufA also cleaves CXCL14/BRAK (breast and kidney-expressed chemokine) at several sites throughout its sequence and, although this attenuates the activities of CXCL14 against both *S. pyogenes* and *F. magna*, killing activity for the latter bacterium is lost much faster than for the former (Frick et al., 2011). Adding to the survival mechanism against chemokines, *F. magna* and *S. pyogenes* can secrete FAF (*F. magna* adhesion factor) and SIC (streptococcal inhibitor of complement), respectively, which directly bind to CXCL14 and CXCL9 to inactivate them without a need for proteolysis.

Bacteria also have ways of using host chemokines to their advantage. For example, MRSA release the virulence factor SPA (*S. aureus* protein A) upon being presented with a majority of chemokines (Yung et al., 2011). This occurs through direct chemokine interactions with MRSA at low bacterial densities, however, the molecular mechanisms leading to SPA release are currently unknown. Furthermore, whether or not a chemokine can induce the release of SPA does not seem to be related to its anti-MRSA activity.

Finally, a specific target has been suggested as part of the antimicrobial mechanism of CXCL10. This protein was identified in *B. anthracis* as FtsX, the transmembrane domain of a putative ATP-binding cassette transporter (Crawford et al., 2011). When incubated with an FtsX-deletion mutant strain of *B. anthracis*, CXCL10 is unable to localize to the cell membrane, thereby conferring resistance to the bacterium. FtsX is well conserved in many bacteria and so it could be an important molecular target for other chemokines and perhaps for some AMPs as well.

## Concluding remarks

Chemokines have long been known for their roles in homeostasis and they play a major role during times of inflammation in the human body (Zlotnik and Yoshie, 2012). Their secondary ability to directly kill microbes was discovered only a decade ago and currently, at least some degree of antimicrobial activity has been found for nearly all human chemokines. While some may argue that these results may not hold much weight outside of the forgiving conditions that can be created inside the laboratory, this secondary “moonlighting” function seems crucial for the immune system *in vivo* (Wolf and Moser, 2012). Much like AMPs, these chemokines have a large cationic surface to promote electrostatic interactions with bacterial surfaces while keeping them harmless to eukaryotic cells. The antimicrobial activity can be modulated by partial digestion, conformational rearrangements, self-association, and through molecular interactions, particularly with GAGs. The structural features that are responsible for distinguishing the potency and range of activity of one chemokine from another is still unclear. To gain a better understanding of their behavior *in vivo*, future structural work in the chemokine research field should take into account their antimicrobial functions in addition to their signaling activities.

### Conflict of interest statement

The authors declare that the research was conducted in the absence of any commercial or financial relationships that could be construed as a potential conflict of interest.

## Abbreviations

AMP: antimicrobial peptide
BRAK: breast and kidney-expressed chemokine
CCL: CC chemokine ligand
CD: circular dichroism
CXCL: CXC chemokine ligand
CXCR: CXC chemokine receptor
CTAP-3: connective tissue-activating peptide 3
FAF: *F. magna* adhesion factor
GAG: glycosaminoglycan
GCP-2: granulocyte chemotactic protein 2
HBD: human β-defensins
His-5: histatin-5
HNP: human neutrophil protein
IL-8: interleukin-8
IP-10: interferon γ-induced protein-10
I-TAC: interferon-inducible T-cell α chemoattractant
mCCL: murine CCL
MCP: monocyte chemoattractant protein
MIG: monokine induced by γ-interferon
MIP-3α: macrophage-inflammatory protein-3α
MRSA: methicillin-resistant *S. aureus*
MSSA: methicillin-sensitive *S. aureus*
NAP-2: neutrophil-activating peptide 2
NMR: nuclear magnetic resonance
NOE: nuclear Overhauser effect
PBP: platelet basic protein
PF-4: platelet factor 4
RANTES: regulated-upon activation, normal T-cell expressed, and secreted
SDF-1α: stromal cell-derived factor-1α
SIC: streptococcal inhibitor of complement
SPA: *S. aureus* protein A
TC-1: thrombocidin-1.

## References

[B1] Allen S. J. Crown S. E. Handel T. M. (2007). Chemokine: receptor structure, interactions, and antagonism. Annu. Rev. Immunol. 25, 787–820 10.1146/annurev.immunol.24.021605.090529 17291188

[B2] Arias M. Zaat S. A. Vogel H. J. (2012). Structure-function relationships of antimicrobial chemokines, in Antimicrobial Peptides and Innate Immunity, eds Hiemstra P. Zaat S. A. (Springer) (in press).

[B3] Auvynet C. El Amri C. Lacombe C. Bruston F. Bourdais J. Nicolas P. (2008). Structural requirements for antimicrobial versus chemoattractant activities for dermaseptin S9. FEBS J. 275, 4134–4151 10.1111/j.1742-4658.2008.06554.x 18637027

[B4] Balkwill F. R. (2012). The chemokine system and cancer. J. Pathol. 226, 148–157 10.1002/path.3029 21989643

[B5] Bjorstad A. Fu H. Karlsson A. Dahlgren C. Bylund J. (2005). Interleukin-8-derived peptide has antibacterial activity. Antimicrob. Agents Chemother. 49, 3889–3895 10.1128/AAC.49.9.3889-3895.2005 16127067 PMC1195386

[B6] Bourbigot S. Dodd E. Horwood C. Cumby N. Fardy L. Welch W. H. (2009). Antimicrobial peptide RP-1 structure and interactions with anionic versus zwitterionic micelles. Biopolymers 91, 1–13 10.1002/bip.21071 18712851

[B7] Bowdish D. M. Davidson D. J. Scott M. G. Hancock R. E. (2005). Immunomodulatory activities of small host defense peptides. Antimicrob. Agents Chemother. 49, 1727–1732 15855488 10.1128/AAC.49.5.1727-1732.2005 PMC1087655

[B8] Brandt E. Ludwig A. Petersen F. Flad H. D. (2000). Platelet-derived CXC chemokines: old players in new games. Immunol. Rev. 177, 204–216 10.1034/j.1600-065X.2000.17705.x 11138777

[B9] Brown K. L. Hancock R. E. (2006). Cationic host defense (antimicrobial) peptides. Curr. Opin. Immunol. 18, 24–30 10.1016/j.coi.2005.11.004 16337365

[B10] Burkhardt A. M. Tai K. P. Flores-Guiterrez J. P. Vilches-Cisneros N. Kamdar K. Barbosa-Quintana O. (2012). CXCL17 is a mucosal chemokine elevated in idiopathic pulmonary fibrosis that exhibits broad antimicrobial activity. J. Immunol. 188, 6399–6406 10.4049/jimmunol.1102903 22611239 PMC3370106

[B11] Chan D. I. Hunter H. N. Tack B. F. Vogel H. J. (2008). Human macrophage inflammatory protein 3alpha: protein and peptide nuclear magnetic resonance solution structures, dimerization, dynamics, and anti-infective properties. Antimicrob. Agents Chemother. 52, 883–894 10.1128/AAC.00805-07 18086840 PMC2258517

[B12] Clore G. M. Appella E. Yamada M. Matsushima K. Gronenborn A. M. (1990). Three-dimensional structure of interleukin 8 in solution. Biochemistry 29, 1689–1696 2184886 10.1021/bi00459a004

[B13] Collin M. Linge H. M. Bjartell A. Giwercman A. Malm J. Egesten A. (2008). Constitutive expression of the antibacterial CXC chemokine GCP-2/CXCL6 by epithelial cells of the male reproductive tract. J. Reprod. Immunol. 79, 37–43 10.1016/j.jri.2008.08.003 18809212

[B14] Crawford M. A. Lowe D. E. Fisher D. J. Stibitz S. Plaut R. D. Beaber J. W. (2011). Identification of the bacterial protein FtsX as a unique target of chemokine-mediated antimicrobial activity against *Bacillus anthracis*. Proc. Natl. Acad. Sci. U.S.A. 108, 17159–17164 10.1073/pnas.1108495108 21949405 PMC3193227

[B15] Davis D. A. Singer K. E. De La Luz Sierra M. Narazaki M. Yang F. Fales H. M. (2005). Identification of carboxypeptidase N as an enzyme responsible for C-terminal cleavage of stromal cell-derived factor-1alpha in the circulation. Blood 105, 4561–4568 10.1182/blood-2004-12-4618 15718415 PMC1895000

[B16] Dawson R. M. Liu C. Q. (2010). Disulphide bonds of the peptide protegrin-1 are not essential for antimicrobial activity and haemolytic activity. Int. J. Antimicrob. Agents 36, 579–580 10.1016/j.ijantimicag.2010.08.011 20947309

[B17] Dempsey C. E. Ueno S. Avison M. B. (2003). Enhanced membrane permeabilization and antibacterial activity of a disulfide-dimerized magainin analogue. Biochemistry 42, 402–409 10.1021/bi026328h 12525167

[B18] Denis C. Deiteren K. Mortier A. Tounsi A. Fransen E. Proost P. (2012). C-Terminal clipping of chemokine CCL1/I-309 enhances CCR8-Mediated intracellular calcium release and anti-apoptotic activity. PLoS ONE 7:e34199 10.1371/journal.pone.0034199 22479563 PMC3313992

[B19] Dominguez C. Boelens R. Bonvin A. M. (2003). HADDOCK: a protein-protein docking approach based on biochemical or biophysical information. J. Am. Chem. Soc. 125, 1731–1737 10.1021/ja026939x 12580598

[B20] Drury L. J. Ziarek J. J. Gravel S. Veldkamp C. T. Takekoshi T. Hwang S. T. (2011). Monomeric and dimeric CXCL12 inhibit metastasis through distinct CXCR4 interactions and signaling pathways. Proc. Natl. Acad. Sci. U.S.A. 108, 17655–17660 10.1073/pnas.1101133108 21990345 PMC3203819

[B21] Egesten A. Eliasson M. Johnsson H. M. Olin A. I. Morgelin M. Mueller A. (2007). The CXC chemokine MIG/CXCL9 is important in innate immunity against streptococcus pyogenes. J. Infect. Dis. 195, 684–693 10.1086/510857 17262710

[B22] Egesten A. Olin A. I. Linge H. M. Yadav M. Morgelin M. Karlsson A. (2009). SpeB of Streptococcus pyogenes differentially modulates antibacterial and receptor activating properties of human chemokines. PLoS ONE 4:e4769 10.1371/journal.pone.0004769 19274094 PMC2652026

[B23] Ehlert J. E. Gerdes J. Flad H. D. Brandt E. (1998). Novel C-terminally truncated isoforms of the CXC chemokine beta-thromboglobulin and their impact on neutrophil functions. J. Immunol. 161, 4975–4982 9794434

[B24] Esche C. Stellato C. Beck L. A. (2005). Chemokines: key players in innate and adaptive immunity. J. Invest. Dermatol. 125, 615–628 10.1111/j.0022-202X.2005.23841.x 16185259

[B25] Fernandez E. J. Lolis E. (2002). Structure, function, and inhibition of chemokines. Annu. Rev. Pharmacol. Toxicol. 42, 469–499 11807180 10.1146/annurev.pharmtox.42.091901.115838

[B26] Frick I. M. Nordin S. L. Baumgarten M. Morgelin M. Sorensen O. E. Olin A. I. (2011). Constitutive and inflammation-dependent antimicrobial peptides produced by epithelium are differentially processed and inactivated by the commensal Finegoldia magna and the pathogen *Streptococcus pyogenes*. J. Immunol. 187, 4300–4309 10.4049/jimmunol.1004179 21918193

[B27] Gandhi N. S. Mancera R. L. (2011). Molecular dynamics simulations of CXCL-8 and its interactions with a receptor peptide, heparin fragments, and sulfated linked cyclitols. J. Chem. Inf. Model. 51, 335–358 10.1021/ci1003366 21299226

[B28] Gusman H. Lendenmann U. Grogan J. Troxler R. F. Oppenheim F. G. (2001). Is salivary histatin 5 a metallopeptide? Biochim. Biophys. Acta 1545, 86–95 10.1016/S0167-4838(00)00265-X 11342034

[B29] Haney E. F. Hunter H. N. Matsuzaki K. Vogel H. J. (2009). Solution NMR studies of amphibian antimicrobial peptides: linking structure to function? Biochim. Biophys. Acta 1788, 1639–1655 10.1016/j.bbamem.2009.01.002 19272309

[B30] Haney E. F. Nathoo S. Vogel H. J. Prenner E. J. (2010). Induction of non-lamellar lipid phases by antimicrobial peptides: a potential link to mode of action. Chem. Phys. Lipids 163, 82–93 10.1016/j.chemphyslip.2009.09.002 19799887

[B31] Hasan L. Mazzucchelli L. Liebi M. Lis M. Hunger R. E. Tester A. (2006). Function of liver activation-regulated chemokine/CC chemokine ligand 20 is differently affected by cathepsin B and cathepsin D processing. J. Immunol. 176, 6512–6522 16709808 10.4049/jimmunol.176.11.6512

[B32] Hensbergen P. J. Verzijl D. Balog C. I. Dijkman R. Van Der Schors R. C. Van Der Raaij-Helmer E. M. (2004). Furin is a chemokine-modifying enzyme: *in vitro* and *in vivo* processing of CXCL10 generates a C-terminally truncated chemokine retaining full activity. J. Biol. Chem. 279, 13402–13411 10.1074/jbc.M312814200 14739277

[B33] Hieshima K. Ohtani H. Shibano M. Izawa D. Nakayama T. Kawasaki Y. (2003). CCL28 has dual roles in mucosal immunity as a chemokine with broad-spectrum antimicrobial activity. J. Immunol. 170, 1452–1461 12538707 10.4049/jimmunol.170.3.1452

[B34] Hileman R. E. Fromm J. R. Weiler J. M. Linhardt R. J. (1998). Glycosaminoglycan-protein interactions: definition of consensus sites in glycosaminoglycan binding proteins. Bioessays 20, 156–167 10.1002/(SICI)1521-1878(199802)20:2<156::AID-BIES8>3.0.CO;2-R 9631661

[B35] Jang W. S. Bajwa J. S. Sun J. N. Edgerton M. (2010). Salivary histatin 5 internalization by translocation, but not endocytosis, is required for fungicidal activity in *Candida albicans*. Mol. Microbiol. 77, 354–370 10.1111/j.1365-2958.2010.07210.x 20487276 PMC2909388

[B36] Jansma A. L. Kirkpatrick J. P. Hsu A. R. Handel T. M. Nietlispach D. (2010). NMR analysis of the structure, dynamics, and unique oligomerization properties of the chemokine CCL27. J. Biol. Chem. 285, 14424–14437 10.1074/jbc.M109.091108 20200157 PMC2863231

[B37] Keizer D. W. Crump M. P. Lee T. W. Slupsky C. M. Clark-Lewis I. Sykes B. D. (2000). Human CC chemokine I-309, structural consequences of the additional disulfide bond. Biochemistry 39, 6053–6059 10.1021/bi000089l 10821677

[B38] Kiefer F. Siekmann A. F. (2011). The role of chemokines and their receptors in angiogenesis. Cell. Mol. Life Sci. 68, 2811–2830 10.1007/s00018-011-0677-7 21479594 PMC11115067

[B39] Kim K. S. Rajarathnam K. Clark-Lewis I. Sykes B. D. (1996). Structural characterization of a monomeric chemokine: monocyte chemoattractant protein-3. FEBS Lett. 395, 277–282 10.1016/0014-5793(96)01024-1 8898111

[B40] Kofuku Y. Yoshiura C. Ueda T. Terasawa H. Hirai T. Tominaga S. (2009). Structural basis of the interaction between chemokine stromal cell-derived factor-1/CXCL12 and its G-protein-coupled receptor CXCR4. J. Biol. Chem. 284, 35240–35250 10.1074/jbc.M109.024851 19837984 PMC2787383

[B41] Krijgsveld J. Zaat S. A. Meeldijk J. Van Veelen P. A. Fang G. Poolman B. (2000). Thrombocidins, microbicidal proteins from human blood platelets, are C-terminal deletion products of CXC chemokines. J. Biol. Chem. 275, 20374–20381 10.1074/jbc.275.27.20374 10877842

[B42] Kwakman P. H. Krijgsveld J. De Boer L. Nguyen L. T. Boszhard L. Vreede J. (2011). Native thrombocidin-1 and unfolded thrombocidin-1 exert antimicrobial activity via distinct structural elements. J. Biol. Chem. 286, 43506–43514 10.1074/jbc.M111.248641 22025617 PMC3234844

[B43] Laguri C. Sapay N. Simorre J. P. Brutscher B. Imberty A. Gans P. (2011). 13C-labeled heparan sulfate analogue as a tool to study protein/heparan sulfate interactions by NMR spectroscopy: application to the CXCL12alpha chemokine. J. Am. Chem. Soc. 133, 9642–9645 10.1021/ja201753e 21634378

[B44] Lehner T. Wang Y. Whittall T. Seidl T. (2011). Innate immunity and HIV-1 infection. Adv. Dent. Res. 23, 19–22 21441475 10.1177/0022034511399081

[B45] Linge H. M. Collin M. Nordenfelt P. Morgelin M. Malmsten M. Egesten A. (2008). The human CXC chemokine granulocyte chemotactic protein 2 (GCP-2)/CXCL6 possesses membrane-disrupting properties and is antibacterial. Antimicrob. Agents Chemother. 52, 2599–2607 10.1128/AAC.00028-08 18443119 PMC2443903

[B46] Liu B. Wilson E. (2010). The antimicrobial activity of CCL28 is dependent on C-terminal positively-charged amino acids. Eur. J. Immunol. 40, 186–196 10.1002/eji.200939819 19830739 PMC2866449

[B47] Lohner K. (2001). The role of membrane lipid composition in cell targeting of antimicrobial peptides, in Development of Novel Antimicrobial Agents: Emerging Strategies, ed Lohner K. (Wymondham: Horizon Scientific Press), 149–165

[B48] Lortat-Jacob H. (2009). The molecular basis and functional implications of chemokine interactions with heparan sulphate. Curr. Opin. Struct. Biol. 19, 543–548 10.1016/j.sbi.2009.09.003 19800217

[B49] Malkowski M. G. Wu J. Y. Lazar J. B. Johnson P. H. Edwards B. F. (1995). The crystal structure of recombinant human neutrophil-activating peptide-2 (M6L) at 1.9-A resolution. J. Biol. Chem. 270, 7077–7087 10.1074/jbc.270.13.7077 7706245

[B50] Mani R. Cady S. D. Tang M. Waring A. J. Lehrer R. I. Hong M. (2006). Membrane-dependent oligomeric structure and pore formation of a beta-hairpin antimicrobial peptide in lipid bilayers from solid-state NMR. Proc. Natl. Acad. Sci. U.S.A. 103, 16242–16247 10.1073/pnas.0605079103 17060626 PMC1637567

[B51] Martinez-Becerra F. Silva D. A. Dominguez-Ramirez L. Mendoza-Hernandez G. Lopez-Vidal Y. Soldevila G. (2007). Analysis of the antimicrobial activities of a chemokine-derived peptide (CDAP-4) on *Pseudomonas aeruginosa*. Biochem. Biophys. Res. Commun. 355, 352–358 10.1016/j.bbrc.2007.01.188 17307153

[B52] Mayo K. H. Chen M. J. (1989). Human platelet factor 4 monomer-dimer-tetramer equilibria investigated by 1H NMR spectroscopy. Biochemistry 28, 9469–9478 2611243 10.1021/bi00450a034

[B53] Mihajlovic M. Lazaridis T. (2010). Antimicrobial peptides in toroidal and cylindrical pores. Biochim. Biophys. Acta 1798, 1485–1493 10.1016/j.bbamem.2010.04.004 20403332 PMC2885466

[B54] Mortier A. Gouwy M. Van Damme J. Proost P. (2011). Effect of posttranslational processing on the *in vitro* and *in vivo* activity of chemokines. Exp. Cell Res. 317, 642–654 10.1016/j.yexcr.2010.11.016 21146523

[B55] Mortier A. Van Damme J. Proost P. (2008). Regulation of chemokine activity by posttranslational modification. Pharmacol. Ther. 120, 197–217 10.1016/j.pharmthera.2008.08.006 18793669

[B56] Murphy J. W. Yuan H. Kong Y. Xiong Y. Lolis E. J. (2010). Heterologous quaternary structure of CXCL12 and its relationship to the CC chemokine family. Proteins 78, 1331–1337 10.1002/prot.22666 20077567 PMC3021379

[B57] Nesmelova I. V. Sham Y. Dudek A. Z. Van Eijk L. I. Wu G. Slungaard A. (2005). Platelet factor 4 and interleukin-8 CXC chemokine heterodimer formation modulates function at the quaternary structural level. J. Biol. Chem. 280, 4948–4958 10.1074/jbc.M405364200 15531763

[B58] Nesmelova I. V. Sham Y. Gao J. Mayo K. H. (2008). CXC and CC chemokines form mixed heterodimers: association free energies from molecular dynamics simulations and experimental correlations. J. Biol. Chem. 283, 24155–24166 10.1074/jbc.M803308200 18550532 PMC2527121

[B59] Nguyen L. T. Chan D. I. Boszhard L. Zaat S. A. Vogel H. J. (2010a). Structure-function studies of chemokine-derived carboxy-terminal antimicrobial peptides. Biochim. Biophys. Acta 1798, 1062–1072 10.1016/j.bbamem.2009.11.021 20004172

[B60] Nguyen L. T. Chau J. K. Perry N. A. De Boer L. Zaat S. A. Vogel H. J. (2010b). Serum stabilities of short tryptophan- and arginine-rich antimicrobial peptide analogs. PLoS ONE 5:e12684 10.1371/journal.pone.0012684 20844765 PMC2937036

[B61] Nguyen L. T. Haney E. F. Vogel H. J. (2011a). The expanding scope of antimicrobial peptide structures and their modes of action. Trends Biotechnol. 29, 464–472 10.1016/j.tibtech.2011.05.001 21680034

[B62] Nguyen L. T. Kwakman P. H. Chan D. I. Liu Z. De Boer L. Zaat S. A. (2011b). Exploring platelet chemokine antimicrobial activity: nuclear magnetic resonance backbone dynamics of NAP-2 and TC-1. Antimicrob. Agents Chemother. 55, 2074–2083 10.1128/AAC.01351-10 21321145 PMC3088234

[B63] Nicolas P. (2009). Multifunctional host defense peptides: intracellular-targeting antimicrobial peptides. FEBS J. 276, 6483–6496 10.1111/j.1742-4658.2009.07359.x 19817856

[B64] Pichert A. Samsonov S. A. Theisgen S. Thomas L. Baumann L. Schiller J. (2012). Characterization of the interaction of interleukin-8 with hyaluronan, chondroitin sulfate, dermatan sulfate and their sulfated derivatives by spectroscopy and molecular modeling. Glycobiology 22, 134–145 10.1093/glycob/cwr120 21873605 PMC3230280

[B65] Proudfoot A. E. (2006). The biological relevance of chemokine-proteoglycan interactions. Biochem. Soc. Trans. 34, 422–426 10.1042/BST0340422 16709177

[B66] Raj P. A. Marcus E. Sukumaran D. K. (1998). Structure of human salivary histatin 5 in aqueous and nonaqueous solutions. Biopolymers 45, 51–67 10.1002/(SICI)1097-0282(199801)45:1<51::AID-BIP5>3.0.CO;2-Y 9433185

[B67] Rajagopalan L. Rajarathnam K. (2006). Structural basis of chemokine receptor function–a model for binding affinity and ligand selectivity. Biosci. Rep. 26, 325–339 10.1007/s10540-006-9025-9 17024562 PMC2671010

[B68] Ramamoorthy A. Thennarasu S. Tan A. Gottipati K. Sreekumar S. Heyl D. L. (2006). Deletion of all cysteines in tachyplesin I abolishes hemolytic activity and retains antimicrobial activity and lipopolysaccharide selective binding. Biochemistry 45, 6529–6540 10.1021/bi052629q 16700563 PMC2515376

[B69] Ren M. Guo Q. Guo L. Lenz M. Qian F. Koenen R. R. (2010). Polymerization of MIP-1 chemokine (CCL3 and CCL4) and clearance of MIP-1 by insulin-degrading enzyme. EMBO J. 29, 3952–3966 10.1038/emboj.2010.256 20959807 PMC3020635

[B70] Salanga C. L. Handel T. M. (2011). Chemokine oligomerization and interactions with receptors and glycosaminoglycans: the role of structural dynamics in function. Exp. Cell Res. 317, 590–601 10.1016/j.yexcr.2011.01.004 21223963 PMC3089961

[B71] Schibli D. J. Hunter H. N. Aseyev V. Starner T. D. Wiencek J. M. Mccray P. B. (2002). The solution structures of the human beta-defensins lead to a better understanding of the potent bactericidal activity of HBD3 against Staphylococcus aureus. J. Biol. Chem. 277, 8279–8289 10.1074/jbc.M108830200 11741980

[B72] Schmidtchen A. Frick I. M. Bjorck L. (2001). Dermatan sulphate is released by proteinases of common pathogenic bacteria and inactivates antibacterial alpha-defensin. Mol. Microbiol. 39, 708–713 10.1046/j.1365-2958.2001.02251.x 11169110

[B73] Schroeder B. O. Wu Z. Nuding S. Groscurth S. Marcinowski M. Beisner J. (2011). Reduction of disulphide bonds unmasks potent antimicrobial activity of human β-defensin 1. Nature 469, 419–423 10.1038/nature09674 21248850

[B74] Seo E. S. Blaum B. S. Vargues T. De Cecco M. Deakin J. A. Lyon M. (2010). Interaction of human beta-defensin 2 (HBD2) with glycosaminoglycans. Biochemistry 49, 10486–10495 10.1021/bi1011749 21062008

[B75] Seshadri Sundararajan V. Gabere M. N. Pretorius A. Adam S. Christoffels A. Lehvaslaiho M. (2012). DAMPD: a manually curated antimicrobial peptide database. Nucleic Acids Res. 40, D1108–D1112 10.1093/nar/gkr1063 22110032 PMC3244992

[B76] Severin I. C. Gaudry J. P. Johnson Z. Kungl A. Jansma A. Gesslbauer B. (2010). Characterization of the chemokine CXCL11-heparin interaction suggests two different affinities for glycosaminoglycans. J. Biol. Chem. 285, 17713–17724 10.1074/jbc.M109.082552 20363748 PMC2878535

[B77] Sticht H. Escher S. E. Schweimer K. Forssmann W. G. Rosch P. Adermann K. (1999). Solution structure of the human CC chemokine 2: a monomeric representative of the CC chemokine subtype. Biochemistry 38, 5995–6002 10.1021/bi990065i 10320325

[B78] Swaminathan G. J. Holloway D. E. Colvin R. A. Campanella G. K. Papageorgiou A. C. Luster A. D. (2003). Crystal structures of oligomeric forms of the IP-10/CXCL10 chemokine. Structure 11, 521–532 10.1016/S0969-2126(03)00070-4 12737818

[B79] Tang Y. Q. Yeaman M. R. Selsted M. E. (2002). Antimicrobial peptides from human platelets. Infect. Immun. 70, 6524–6533 10.1128/?IAI.70.12.6524-6533.2002 12438321 PMC132966

[B80] Tencza S. B. Creighton D. J. Yuan T. Vogel H. J. Montelaro R. C. Mietzner T. A. (1999). Lentivirus-derived antimicrobial peptides: increased potency by sequence engineering and dimerization. J. Antimicrob. Chemother. 44, 33–41 10.1093/jac/44.1.33 10459808

[B81] Tsai H. Bobek L. A. (1998). Human salivary histatins: promising anti-fungal therapeutic agents. Crit. Rev. Oral Biol. Med. 9, 480–497 10.1177/10454411980090040601 9825223

[B82] Tuinstra R. L. Peterson F. C. Kutlesa S. Elgin E. S. Kron M. A. Volkman B. F. (2008). Interconversion between two unrelated protein folds in the lymphotactin native state. Proc. Natl. Acad. Sci. U.S.A. 105, 5057–5062 10.1073/pnas.0709518105 18364395 PMC2278211

[B83] Veldkamp C. T. Peterson F. C. Pelzek A. J. Volkman B. F. (2005). The monomer-dimer equilibrium of stromal cell-derived factor-1 (CXCL 12) is altered by pH, phosphate, sulfate, and heparin. Protein Sci. 14, 1071–1081 10.1110/ps.041219505 15741341 PMC2253449

[B84] Wang X. Watson C. Sharp J. S. Handel T. M. Prestegard J. H. (2011). Oligomeric structure of the chemokine CCL5/RANTES from NMR, MS, and SAXS data. Structure 19, 1138–1148 10.1016/j.str.2011.06.001 21827949 PMC3159919

[B85] Weber C. Koenen R. R. (2006). Fine-tuning leukocyte responses: towards a chemokine ‘interactome’. Trends Immunol. 27, 268–273 10.1016/j.it.2006.04.002 16678487

[B86] Wolf M. Albrecht S. Marki C. (2008). Proteolytic processing of chemokines: implications in physiological and pathological conditions. Int. J. Biochem. Cell Biol. 40, 1185–1198 10.1016/j.biocel.2007.12.009 18243768

[B87] Wolf M. Moser B. (2012). Antimicrobial activities of chemokines: not just a side-effect? Front Immunol. 3:213 10.3389/fimmu.2012.00213 22837760 PMC3401835

[B88] Wu Z. Hoover D. M. Yang D. Boulegue C. Santamaria F. Oppenheim J. J. (2003). Engineering disulfide bridges to dissect antimicrobial and chemotactic activities of human beta-defensin 3. Proc. Natl. Acad. Sci. U.S.A. 100, 8880–8885 10.1073/pnas.1533186100 12840147 PMC166407

[B89] Yang D. Chen Q. Hoover D. M. Staley P. Tucker K. D. Lubkowski J. (2003). Many chemokines including CCL20/MIP-3alpha display antimicrobial activity. J. Leukoc. Biol. 74, 448–455 10.1189/jlb.0103024 12949249

[B90] Yang D. Chertov O. Bykovskaia S. N. Chen Q. Buffo M. J. Shogan J. (1999). Beta-defensins: linking innate and adaptive immunity through dendritic and T cell CCR6. Science 286, 525–528 10.1126/science.286.5439.525 10521347

[B91] Yeaman M. R. Yount N. Y. Waring A. J. Gank K. D. Kupferwasser D. Wiese R. (2007). Modular determinants of antimicrobial activity in platelet factor-4 family kinocidins. Biochim. Biophys. Acta 1768, 609–619 10.1016/j.bbamem.2006.11.010 17217910 PMC2827485

[B92] Yoshiura C. Kofuku Y. Ueda T. Mase Y. Yokogawa M. Osawa M. (2010). NMR analyses of the interaction between CCR5 and its ligand using functional reconstitution of CCR5 in lipid bilayers. J. Am. Chem. Soc. 132, 6768–6777 10.1021/ja100830f 20423099

[B93] Yount N. Y. Andres M. T. Fierro J. F. Yeaman M. R. (2007). The gamma-core motif correlates with antimicrobial activity in cysteine-containing kaliocin-1 originating from transferrins. Biochim. Biophys. Acta 1768, 2862–2872 10.1016/j.bbamem.2007.07.024 17916323

[B94] Yung S. C. Parenti D. Murphy P. M. (2011). Host chemokines bind to Staphylococcus aureus and stimulate protein A release. J. Biol. Chem. 286, 5069–5077 10.1074/jbc.M110.195180 21138841 PMC3037618

[B95] Zhang Y. Lu W. Hong M. (2010). The membrane-bound structure and topology of a human alpha-defensin indicate a dimer pore mechanism for membrane disruption. Biochemistry 49, 9770–9782 10.1021/bi101512j 20961099 PMC2992833

[B94a] Zlotnik A. Yoshie O. (2012). The chemokine superfamily revisited. Immunity 36, 705–716 10.1016/j.immuni.2012.05.008 22633458 PMC3396424

